# A Critical Appraisal of Off-Label Use and Repurposing of Statins for Non-Cardiovascular Indications: A Systematic Mini-Update and Regulatory Analysis

**DOI:** 10.3390/jcm14155436

**Published:** 2025-08-01

**Authors:** Anna Artner, Irem Diler, Balázs Hankó, Szilvia Sebők, Romána Zelkó

**Affiliations:** 1 Center of Pharmacology and Drug Research & Development, 1085 Budapest, Hungary; artner.anna@semmelweis.hu (A.A.); or balazs.hanko@kim.gov.hu (B.H.); sebok.szilvia@semmelweis.hu (S.S.); 2 University Pharmacy Department of Pharmacy Administration, Semmelweis University, 1085 Budapest, Hungary; 3 Faculty of Pharmacy, Bezmialem Vakif University, 34093 Istanbul, Turkey; 190301013@bavu.edu.tr

**Keywords:** statins, off-label use, drug repurposing, oncology, infectious disease, simvastatin, mevalonate pathway, regulation

## Abstract

**Background:** Statins exhibit pleiotropic anti-inflammatory, antioxidant, and immunomodulatory effects, suggesting their potential in non-cardiovascular conditions. However, evidence supporting their repurposing remains limited, and off-label prescribing policies vary globally. **Objective:** To systematically review evidence on statin repurposing in oncology and infectious diseases, and to assess Hungarian regulatory practices regarding off-label statin use. **Methods:** A systematic literature search (PubMed, Web of Science, Scopus, ScienceDirect; 2010–May 2025) was conducted using the terms “drug repositioning” OR “off-label prescription” AND “statin” NOT “cardiovascular,” following PRISMA guidelines. Hungarian off-label usage data from the NNGYK (2008–2025) were also analyzed. **Results:** Out of 205 publications, 12 met the inclusion criteria—75% were oncology-focused, and 25% focused on infectious diseases. Most were preclinical (58%); only 25% offered strong clinical evidence. Applications included hematologic malignancies, solid tumors, Cryptococcus neoformans, SARS-CoV-2, and dengue virus. Mechanisms involved mevalonate pathway inhibition and modulation of host immune responses. Hungarian data revealed five approved off-label statin uses—three dermatologic and two pediatric metabolic—supported by the literature and requiring post-treatment reporting. **Conclusions:** While preclinical findings are promising, clinical validation of off-label statin use remains limited. Statins should be continued in cancer patients with cardiovascular indications, but initiation for other purposes should be trial-based. Future directions include biomarker-based personalization, regulatory harmonization, and cost-effectiveness studies.

## 1. Introduction

Statins inhibit hydroxymethyl glutaryl coenzyme A reductase (HMG-CoA reductase), an essential enzyme in the cholesterol biosynthesis pathway. These medications effectively lower low-density lipoprotein cholesterol (LDL-C) and triglyceride levels, while increasing high-density lipoprotein cholesterol (HDL-C) [1]. Beyond their lipid-lowering effects, statins exhibit pleiotropic properties—such as anti-inflammatory, antioxidant, and immunomodulatory activities—that have sparked interest in their potential repurposing for non-cardiovascular diseases [2]. Despite this growing attention, there remains limited regulatory-approved evidence supporting statin use outside of cardiovascular indications [1].

With increasing scientific interest in novel applications, this article focuses on both the off-label use and therapeutic repurposing of statins. Off-label use refers to prescriptions for indications, doses, or dosage forms not approved by regulatory authorities such as the U.S. Food and Drug Administration (FDA). Physicians may prescribe off-label treatments based on clinical judgment and available evidence, weighing potential risks and benefits [3,4]. This is distinct from pleiotropy, which refers to an approved drug exerting multiple pharmacological effects across different biological pathways [2].

Regulatory policies governing off-label drug use vary widely between countries (Table 1) [5,6,7,8,9,10,11]. In the European Union, the European Medicines Agency (EMA) provides general regulatory frameworks, but member states retain discretion over national implementation. For instance, France has established Temporary Recommendations for Use (RTUs) to formally manage off-label prescriptions for public health purposes. In Hungary, off-label drug use is regulated by the 2005 Act XCV and requires approval from the National Institute of Public Health and Pharmacology (NNGYK) [12]. Approval is granted only if the drug is authorized in Hungary or abroad, and if authorized treatments are ineffective or unavailable for the patient’s condition. The application must demonstrate that the off-label use has a realistic potential to benefit the patient. The submission must include thorough documentation citing clinical trials, the professional literature, and relevant guidelines, along with a consent form signed by the patient or their representative, acknowledging the treatment’s off-label status and associated risks. The prescribing physician must also commit to regularly reporting the patient’s progress and any adverse effects to the NNGYK. This process ensures off-label prescribing prioritizes patient safety and treatment efficacy [13]. Hungary utilizes a named-patient authorization system requiring case-by-case approval. In contrast, the Netherlands allows off-label prescribing without prior regulatory approval if it aligns with recognized clinical protocols or professional guidelines [14,15]. These examples illustrate the heterogeneity of off-label regulation across Europe, which has important implications for the broader application and clinical integration of statins.

The aim of this review is to provide an overview of statin use and potential repurposing in non-cardiovascular indications. This mini-update examines the off-label and repositioned uses of statins, emphasizing both the scientific evidence and the regulatory context surrounding their application. This dual focus seeks to bridge clinical innovation with responsible, evidence-based prescribing.

## 2. Materials and Methods

### 2.1. Search Strategy and Eligibility Criteria

**Figure 1:**
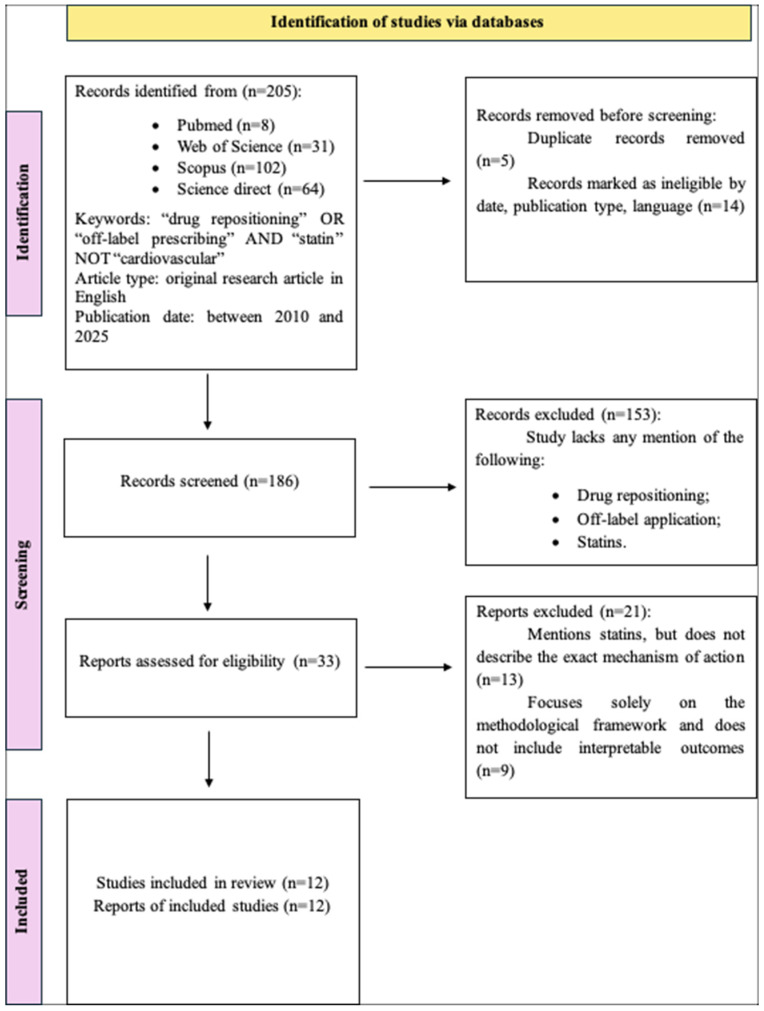
PRISMA flowchart.

The literature search was conducted in accordance with the Preferred Reporting Items for Systematic Reviews and Meta-Analyses (PRISMA) guidelines [16]. Figure 1 illustrates the study selection process using a flow diagram. A comprehensive search was performed across multiple databases, including PubMed, Web of Science, Scopus, and ScienceDirect, to identify original research articles focused on the repositioning or off-label use of statins—specifically their application beyond cholesterol-lowering indications.

To accommodate the variability in how the term off-label is understood, the literature search was based on the following set of keywords: “drug repositioning” OR “off-label prescription” AND “statin” NOT “cardiovascular”, and was limited to studies published between 2010 and May 2025.

A total of 205 studies were identified from the databases, but only 12 met the inclusion criteria. These criteria were as follows: (1) original research articles in English addressing the off-label use or repositioning of statins, and (2) studies involving statin therapy for indications beyond their primary cardiovascular use. Studies were excluded if they were duplicates, lacked information on statin repurposing or off-label use, or focused on statins solely as comedications rather than as the main intervention.

Two independent authors (A.A. and I.D.) screened the records, followed by full-text review of eligible articles. Any discrepancies were resolved through discussion with a third authors (R.Z.).

#### Data Extraction

Data extraction was performed independently by two authors (A.A. and I.D.) using a standardized form. For each included study, the following information was collected: indication, disease category, study design, type of statin, outcome/effect, and additional notes regarding the role of the statin. All extracted data were compiled into a summary table (Table 2) to facilitate comparative analysis and discussion of the results. Any discrepancies were resolved by consensus or, if necessary, through consultation with a third authors (Z.R.).

### 2.2. Hungarian Off-Label Use Data

To investigate the off-label use of statins in Hungary, data were retrieved from an Excel file available on the website of the National Institute for Public Health and Pharmacy (NNGYK) [29]. The dataset spans from 2008 to May 2025 and includes all recorded requests for the off-label use of any statin marketed in Hungary. Search terms such as “statin” and “sztatin” were used to identify relevant entries.

The Excel file provides detailed information on each off-label use request, including the active ingredient, dosage form, strength, requested indication, patient demographics, and decision outcome (approved or rejected). Additional fields, such as the decision date, justification references, and authorization conditions, are also available.

Analysis of this dataset revealed five relevant off-label statin use requests, the details of which are summarized in Table 3.

## 3. Results

**Figure 2:**
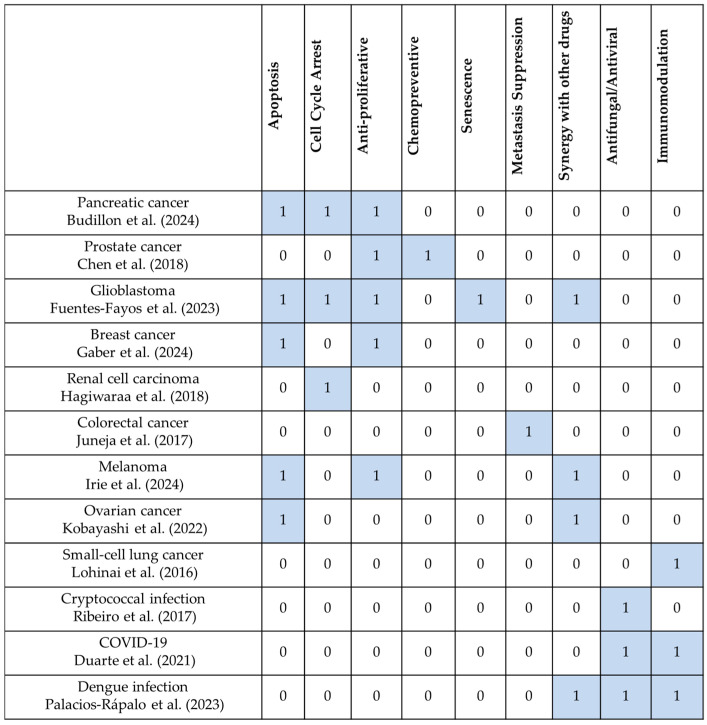
Mechanistic effect types of statins across repurposed non-cardiovascular indications. (Cells marked with “1” indicate that the effect was observed or proposed in the referenced study; cells marked with “0” indicate no direct evidence or mention of that mechanism in the given context) [17,18,19,20,21,22,23,24,25,26,27,28].

A systematic evaluation of the selected studies revealed diverse off-label applications and repurposing strategies for statins in non-cardiovascular indications. The literature was grouped into two therapeutic areas: oncology and infectious diseases (Table 4 and Table 5). Each study was analyzed based on its therapeutic context, study design, type of statin used, observed effects, proposed mechanisms, and clinical relevance. Figure 2 shows the mechanistic themes across indications. Additionally, data on off-label statin use in Hungary were reviewed, offering insight into real-world prescribing practices and regulatory decisions. Together, these findings reflect the growing interest in repositioning statins as multi-target therapeutics.

### 3.1. Statin Repurposing in Oncology: Clinical Trials, Mechanisms, and Translational Insights

In their investigator-initiated, multicenter, open-label Phase II randomized trial, Budillon et al. are currently evaluating a novel combination therapy for metastatic pancreatic ductal adenocarcinoma (mPDAC), a malignancy with a dismal prognosis. Treatment-naive patients with mPDAC are randomized to receive standard first-line chemotherapy—either gemcitabine plus nab-paclitaxel or the more intensive PAXG regimen—with or without the addition of valproic acid (VPA), a histone deacetylase (HDAC) inhibitor, and simvastatin. In the experimental arm, simvastatin is administered orally at a fixed dose of 20 mg/day, starting one week before chemotherapy, while VPA is titrated to achieve therapeutic serum levels. The trial aims to enroll 240 patients (120 per arm) across Italian and Spanish cancer centers. The primary endpoint is progression-free survival (PFS), with the hypothesis that adding VPA and simvastatin could extend the median PFS from approximately 6 to 9 months. Secondary endpoints include overall survival (OS), objective response rate (ORR), disease control rate (DCR), CA 19-9 tumor marker reduction, safety, and quality of life. Recruitment began in mid-2023, with an initial safety run-in phase. This study builds on preclinical evidence showing synergy between HDAC inhibition and statin therapy. VPA reverses epigenetic alterations, while simvastatin inhibits the mevalonate pathway; together, they target cancer stem cells, suppress YAP signaling, and reverse TGF-β–driven epithelial–mesenchymal transition—mechanisms shown to sensitize pancreatic tumors to chemotherapy. Strengths of the trial include its randomized design, its use of two well-characterized, affordable agents with favorable safety profiles, and its planned biomarker analyses. However, as a Phase II open-label study, it remains vulnerable to bias and requires validation in larger, confirmatory trials. While its clinical outcomes have not yet been reported, the protocol of this study is included here to reflect the current state of clinical investigation into statin repurposing in oncology. Its inclusion highlights the rationale and methodological direction of prospective studies in this area [17].

Building on this clinical trial approach, Chen et al. adopted a population-based perspective to examine the effect of statin use on prostate cancer (PCa) mortality using real-world data. The study included over 15,000 men newly diagnosed with PCa between 1999 and 2010, stratified by cumulative statin exposure measured in defined daily doses (DDD). Simvastatin and lovastatin were the most commonly prescribed statins. Patients in the highest-exposure group (>180 DDD/year) showed a significantly lower risk of PCa-specific mortality, with a hazard ratio (HR) of 0.63 (95% CI: 0.56–0.71). This inverse association remained significant after adjusting for age, comorbidities, income, and concomitant medications. The findings offer strong epidemiological support for a dose-dependent protective effect of statins in PCa. While the study did not explore molecular mechanisms directly, it references prior research suggesting that statins may inhibit tumor cell proliferation, induce apoptosis, and disrupt cholesterol-dependent oncogenic signaling pathways such as PI3K/AKT [30,31,32,33,34]. However, confirming statins’ role in reducing PCa mortality will require prospective, large-scale clinical trials and mechanistic studies to clarify the underlying biological effects of statin therapy. Chen et al. conducted a meta-analysis investigating the association between statin use—both pre- and post-treatment—and prostate cancer recurrence and survival. Their findings contribute to the growing body of literature exploring statins as potential adjuvant agents in oncology, rather than solely in a primary prevention context [26].

While Chen et al. focused on epidemiological trends, Fuentes-Fayos et al. combined clinical observations with experimental research to evaluate statins in glioblastoma multiforme (GBM) [24,26]. The study was exploratory, analyzing 85 GBM patients—8 on metformin, 17 on statins, and 9 on both—alongside in vitro and in vivo experiments. All patients underwent surgical resection as primary therapy, with statin and/or metformin therapy initiated pre- or post-operatively as part of their comprehensive treatment regimen. Simvastatin was the primary statin tested experimentally, while patient data also included atorvastatin and rosuvastatin. Glioblastoma cell lines (U-87MG, U-118MG), patient-derived GBM cultures, normal human astrocytes, and mouse astrocyte progenitors were treated with metformin (10 mM) and/or simvastatin (10 μM). In mice bearing U-87MG xenografts, combination therapy was administered intratumorally. Both drugs individually inhibited proliferation, migration, tumor-sphere and colony formation, and VEGF secretion, while inducing apoptosis and senescence. These effects were significantly amplified when used in combination. In mice, the dual treatment suppressed tumor growth and enhanced apoptosis. Clinically, patients on both agents showed a trend toward improved median overall survival (~15.5 months) compared to those not receiving either (~12–13 months), although the small sample size precluded statistical significance. Given their ability to cross the blood–brain barrier, lipophilic statins such as simvastatin and atorvastatin may provide superior tissue penetration in glioblastoma. This pharmacokinetic advantage supports their potential utility in treating central nervous system tumors, where drug delivery is often restricted by the intact blood–brain barrier [35,36]. Mechanistically, the synergistic antitumor activity was linked to AKT inhibition and TGFβ activation, triggering cellular senescence and a Senescence-Associated Secretory Phenotype (SASP). The combination also disrupted RNA processing and downregulated oncogenic pathways, including AKT, JAK–STAT, and NFκB. While the findings highlight promising biological effects and potential clinical relevance, the study’s small patient subgroups and preclinical emphasis limit generalizability. The results remain hypothesis-generating and warrant further investigation in larger, controlled clinical studies [24].

Expanding the scope to breast cancer, Gaber et al. explored a nanoparticle-based formulation of atorvastatin to enhance anticancer efficacy both in vitro and in vivo. The drug was formulated into solid lipid nanoparticles (SLNs), with some variants decorated with lactoferrin (Lf) to enhance targeting of MCF-7 breast cancer cells and Ehrlich carcinoma tumors in mice. Solid lipid nanoparticles (SLNs) are lipid-based submicron colloidal carriers composed of physiological lipids that remain solid at body temperature, stabilized by surfactants [37]. In cell cultures, Lf-SLNs exhibited significantly greater cytotoxicity, with IC_50_ values approximately 2–2.5 times lower than those of free atorvastatin or plain SLNs. In vivo, daily intraperitoneal administration of atorvastatin (5–10 mg/kg) via SLNs led to a notable tumor volume reduction and increased caspase-3–mediated apoptosis, with Lf-SLNs producing the strongest effect. Mechanistically, atorvastatin acts by inhibiting HMG-CoA reductase, thereby disrupting the mevalonate pathway, which reduces cholesterol and isoprenoid synthesis. This impairs oncogenic signaling through pathways such as AKT and Rho GTPases and promotes apoptosis. The Lf coating enhances tumor cell uptake, further improving therapeutic efficacy. The study effectively integrates drug formulation and preclinical testing, offering a strong proof of concept for repurposing atorvastatin in oncology. Its strengths include the dual-model design, use of appropriate controls, and quantitative assays for uptake and apoptosis. However, limitations include the use of a single-tumor model, non-physiological intraperitoneal delivery, a lack of systemic toxicity and metastasis data, and uncertain translatability to human oral dosing. While the findings are promising, additional studies—including pharmacokinetics, toxicity profiling, and clinical trials—are essential before considering off-label atorvastatin use in breast cancer treatment [18].

Similarly aiming to enhance existing cancer therapies, Hagiwara et al. investigated how statins might augment the effects of mammalian target of rapamycin (mTOR) inhibitors in renal cell carcinoma. Although the mTOR pathway is named after rapamycin, the study specifically evaluated the mTOR inhibitor everolimus, a rapamycin analog, in combination with statin use. In a retrospective cohort study, RCC patients receiving the mTOR inhibitor everolimus had significantly longer progression-free survival (PFS) if they were regular statin users (median 7.5 vs. 3.2 months). To explore the mechanism, in vitro experiments were performed on RCC cell lines, and in vivo studies used mouse xenografts, with simvastatin administered alongside everolimus. The combination therapy more effectively suppressed tumor cell proliferation and growth than either agent alone. Mechanistically, simvastatin inhibited HMG-CoA reductase, impairing KRAS and Rac1 prenylation. This led to retinoblastoma (Rb) protein activation (hypophosphorylation), inducing G1 cell cycle arrest and sensitizing tumors to everolimus. This translational study is notable for bridging real-world evidence and mechanistic insights, showing how a widely used cardiovascular drug may augment cancer therapy. Strengths include the alignment of clinical and experimental findings, clear identification of Rb as a mechanistic target, and validation in multiple RCC models. Limitations include the retrospective design, potential confounders in statin use, and reliance on preclinical models that may not fully reflect human tumors. Still, the data support further investigation of statins as adjuvants to mTOR inhibitors in RCC, ideally in prospective trials, to confirm their benefits and optimize dosing [27].

The role of statins in suppressing metastatic signaling was further explored by Juneja et al., who targeted the metastasis-associated gene MACC1 in colorectal cancer. Using a combined in vitro and in vivo approach, a high-throughput screen identified lovastatin as a potent inhibitor of MACC1 promoter activity in CRC cell lines (HCT116, SW480). Lovastatin at 5–20 µM downregulated MACC1 mRNA and protein in a dose-dependent manner, reducing cell proliferation, migration, and invasion. In vivo, daily intraperitoneal injections of lovastatin (10 mg/kg for three weeks) in mice with MACC1-overexpressing CRC xenografts significantly suppressed tumor growth and liver metastases. Mechanistically, lovastatin inhibited HMG-CoA reductase, disrupting isoprenoid synthesis and likely affecting prenylated small GTPases and transcription factors that regulate MACC1, thereby blocking its pro-metastatic signaling. This translational study highlights how a well-tolerated statin can modulate a specific oncogene. Strengths include the integration of genetic and pharmacological models, robust metastasis assays, and consistent in vitro and in vivo results. Limitations include the lack of clinical or epidemiologic data, focus on a single gene target, and use of intraperitoneal dosing, which may not align with human pharmacokinetics. Overall, while the findings are promising, clinical validation and pharmacokinetic studies are needed before considering off-label statin use in CRC [21].

In contrast to prior studies focusing on a single agent, Irie et al. investigated the synergistic effects of combining atorvastatin with dipyridamole in melanoma cell lines. Using both human (A375, SK-MEL-28) and spontaneous canine melanoma models, they tested atorvastatin (0.1–10 µM) in combination with dipyridamole (1–3 µM) and observed strong synergistic antiproliferative effects. Notably, the combination of vemurafenib and atorvastatin produced growth inhibition comparable to vemurafenib alone. However, the addition of dipyridamole to this regimen (vemurafenib + atorvastatin + dipyridamole) resulted in enhanced antiproliferative activity, suggesting that the therapeutic benefit is primarily driven by the interaction between atorvastatin and dipyridamole, rather than by atorvastatin monotherapy. Mechanistically, dipyridamole inhibited the sterol regulatory element-binding protein 2 (SREBP2)-mediated feedback upregulation of HMG-CoA reductase (HMGCR), sustaining mevalonate pathway inhibition and amplifying apoptosis. Western blotting showed reduced HMGCR and increased cleaved PARP, while flow cytometry confirmed enhanced apoptosis under combined treatment. This mechanistic study demonstrates how dual targeting of cholesterol biosynthesis and its regulatory feedback potentiates statin-induced cytotoxicity. Strengths include the use of genetically diverse melanoma models and synergy with both dipyridamole and vemurafenib. However, the absence of in vivo data, pharmacokinetic assessments, and reliance on high in vitro doses limit translational relevance. While the findings support further investigation of statin–dipyridamole therapy in melanoma, in vivo validation and dose optimization are essential before considering clinical application [19].

Kobayashi et al. expanded on this combination strategy by incorporating biomarker analysis to guide statin use in ovarian cancer, underscoring a precision medicine approach. Their translational study combined in vitro profiling of human ovarian cancer cell lines (e.g., SK-OV-3, OVCAR-3) with ex vivo histoculture drug response assays using patient tumor samples. Simvastatin (1–10 µM) was the primary statin evaluated. Key findings included the identification of VDAC1 (positive) and LDLRAP1 (negative) as biomarkers correlating with statin sensitivity, and evidence that combining simvastatin with paclitaxel or the histone deacetylase inhibitor (HDACi) panobinostat enhanced antiproliferative effects and apoptosis. Mechanistically, simvastatin inhibited the mevalonate pathway, disrupting GTPase prenylation and inducing apoptosis. Panobinostat amplified this effect via epigenetic activation of pro-apoptotic genes. These results support a precision medicine approach, using biomarkers to predict response and combining therapies to overcome resistance. The study’s strengths lie in its dual use of cell lines and patient-derived tissues, robust biomarker analysis, and clinically relevant drug combinations. However, limitations include its preclinical nature, its lack of in vivo or clinical data, and the uncertain translatability of in vitro simvastatin doses to human therapy. Validation of VDAC1 and LDLRAP1 in clinical trials is needed before off-label use of statins in ovarian cancer can be considered [20].

Finally, Lohinai et al. contributed a real-world clinical perspective, evaluating statin use across a large cohort of small-cell lung cancer (SCLC) patients, reinforcing the potential survival benefit of statin therapy in oncology. Reviewing records from 876 stage 4 SCLC patients at a Hungarian center, they compared overall survival (OS) between regular users and non-users of repositioned agents. Statin use—mainly atorvastatin or simvastatin at standard cardiovascular doses—was associated with a significant OS benefit: the median OS was 8.4 months in statin users vs. 6.1 months in non-users, reflecting a 37% survival increase. Although no mechanistic experiments were included, the authors proposed that statins’ inhibition of HMG-CoA reductase and downstream prenylation pathways may enhance chemotherapy sensitivity or modulate tumor-related inflammation. This study offers real-world clinical evidence suggesting a survival benefit from statins in SCLC. Strengths include a large cohort, careful adjustment for confounders (e.g., age, performance status, radiotherapy), and multivariate analysis. However, as a retrospective, single-center study, it carries limitations such as potential selection bias, a lack of detailed data on statin dosing and adherence, and no mechanistic validation. Nonetheless, the findings support prospective trials and biological studies to assess whether off-label statin use could serve as a low-cost adjunct to standard SCLC therapy [28].

### 3.2. Statins in Infectious Diseases: Antifungal and Antiviral Potential

Ribeiro et al. explored the antifungal potential of atorvastatin against Cryptococcus neoformans, a life-threatening fungal pathogen, especially in immunocompromised patients. Using both in vitro and in vivo models, they evaluated atorvastatin alone and in combination with fluconazole. In vitro, atorvastatin inhibited multiple *C. neoformans* strains at micromolar concentrations. In vivo, BALB/c mice infected intratracheally and treated orally with atorvastatin (10 mg/kg/day) showed a significantly reduced pulmonary fungal burden. Co-administration with fluconazole further enhanced its antifungal efficacy, suggesting a synergy between the two substances. The proposed mechanism involves inhibition of HMG-CoA reductase, impairing ergosterol biosynthesis, which is essential for fungal cell membrane integrity. This translational study supports the off-label use of atorvastatin as an adjunctive antifungal therapy. Its strengths lie in the combined experimental design and demonstrated in vivo efficacy. Its limitations include limited mechanistic validation, a lack of clinical data, and uncertain dosing translatability to humans. Still, the findings offer a strong rationale for further investigation of statins in antifungal strategies [22].

Shifting from fungal infections to viral respiratory illness, Duarte et al. investigated drug repurposing candidates for COVID-19 using a multimodal approach that integrated in silico predictions with experimental validation in a human lung organoid model. The study focused on SARS-CoV-2 infection and combined network-based computational screening, transcriptional profiling, and in vitro testing. Among the evaluated FDA-approved drugs, atorvastatin showed promising inhibition of viral entry. Human stem cell-derived alveolar type II lung organoids were infected with a pseudotyped virus mimicking SARS-CoV-2 spike-mediated entry, and atorvastatin, tested at micromolar concentrations, significantly blocked viral entry without cytotoxicity. Mechanistically, atorvastatin may disrupt viral uptake by modifying cholesterol-rich lipid rafts or through pleiotropic effects on host pathways involved in replication and inflammation. This translational study effectively bridges computational predictions with physiologically relevant models. Its strengths include the innovative use of lung organoids and a data-driven screening pipeline. However, its limitations involve the absence of in vivo or clinical validation, the use of a pseudovirus instead of live SARS-CoV-2, and no pharmacokinetic data on the tested dose. While atorvastatin’s antiviral activity is encouraging, further preclinical and clinical studies are needed to support its off-label use for COVID-19 [25].

Expanding the scope to vector-borne viral infections, Palacios-Rápalo et al. investigated the antiviral potential of atorvastatin, alone and in combination with ivermectin, against dengue virus (DENV) using both in vitro and in vivo models. Human Huh-7 liver cells were used for in vitro assays, with atorvastatin applied at 10–20 µM concentrations. In vivo experiments employed AG129 immunodeficient mice treated orally with atorvastatin (20 mg/kg) and/or ivermectin (4 mg/kg), starting two days prior to infection and continuing for five days. Atorvastatin monotherapy significantly reduced DENV replication in vitro and lowered viral loads in vivo. Its combination with ivermectin produced additive effects, including enhanced viral suppression, reduced levels of inflammatory cytokines (IL-6 and TNF-α), and improved survival in infected mice. Mechanistically, atorvastatin disrupted nuclear transport by downregulating importins KPNA2 and KPNB1, thereby blocking the nuclear import of DENV nonstructural protein 5 (NS5), which is essential for viral replication and immune evasion. This translational study provides robust preclinical evidence for repurposing statins in infectious disease treatment. Key strengths of the study include its evaluation of both monotherapy and combination therapy, its provision of mechanistic insights into host–pathogen interactions, and its use of relevant cell and animal models. Its limitations include its reliance on immunodeficient mice, absence of pharmacokinetic and clinical data, and technical constraints in assessing the nuclear transport of viral proteins like NS3 in vivo [23].

### 3.3. National Experience with Off-Label Statin Use in Hungary

Between 2012 and 2025, five individual off-label authorizations were granted in Hungary for the use of simvastatin in non-standard indications, demonstrating its potential beyond conventional lipid-lowering applications (Table 3). While these cases provide minimal scientific evidence for statin efficacy in non-cardiovascular indications, they illustrate the practical regulatory framework governing off-label prescribing in Hungary. In 2012, a 4-year-old female patient with hypercholesterinaemia was prescribed simvastatin 5 mg daily, titrated up to 20 mg/day for continuous use. The request was approved based on expert opinion and the literature supporting its use in pediatric dyslipidemia, with a requirement to submit an evaluation report upon treatment completion. In 2017, simvastatin was authorized for a 1.5-month-old male infant with Smith–Lemli–Opitz syndrome at a dose of 0.5 mg/kg/day. Evidence from the literature supported its use in congenital metabolic disorders, and regular three-month reporting to the authority was mandated. Another case in 2021 involved a 64-year-old male diagnosed with actinic porokeratosis. Here, simvastatin was formulated as a topical ointment and applied twice daily for three months. The request, supported by the literature, required submission of detailed clinical and safety monitoring data post-treatment. In 2024 and 2025, two further cases involved rare dermatological conditions: a 49-year-old male with superficial disseminated porokeratosis treated with a 2% simvastatin cream once daily, and a 55-year-old female with actinic porokeratosis who used a topical formulation applied twice daily. Both were approved based on published data, with regulatory conditions requiring comprehensive post-treatment evaluation reports to the National Institute of Public Health and Pharmacy (NNGYK). These authorizations reflect applications in rare diseases for which limited therapeutic alternatives exist, consistent with appropriate off-label prescribing principles. These cases illustrate the cautious but evidence-based Hungarian regulatory approach to statin repurposing in both pediatric and adult populations.

## 4. Discussion

### 4.1. Synthesis of Current Evidence

The systematic evaluation of statin repurposing across non-cardiovascular indications reveals a complex landscape of preclinical promise tempered by limited high-quality clinical evidence. The majority of the identified studies (58%) represent early-stage preclinical or in vitro investigations, while only 25% constitute high-level evidence from randomized controlled trials or large population-based studies [38]. Recent clinical developments have provided compelling evidence for the benefits of statin use in specific cancer types, particularly hematologic malignancies. The pooled analysis of 1467 patients with chronic lymphocytic leukemia and small lymphocytic lymphoma demonstrated a remarkable 61% reduction in cancer-specific mortality among statin users, with consistent benefits across different treatment regimens, including ibrutinib-based therapies [39].

### 4.2. Mechanistic Foundations and Pleiotropic Effects

The therapeutic potential of statins beyond cardiovascular disease stems from their multifaceted impact on cellular metabolism and signaling pathways [40]. Inhibition of HMG-CoA reductase disrupts the mevalonate pathway, affecting not only cholesterol synthesis, but also the production of isoprenoid intermediates that are essential for protein prenylation [41]. This mechanism particularly impacts small GTPases, including Ras, Rho, and Rac, whose proper membrane localization and function depend on isoprenylation [42]. The pleiotropic effects of statins encompass anti-inflammatory properties, immunomodulation, and direct antitumor activities [2]. Recent network pharmacology analyses have revealed that statins modulate complex molecular networks extending beyond the mevalonate pathway [43]. Transcriptomic profiling of statin-treated cancer cells demonstrates downregulation of genes involved in cell cycle progression, DNA repair, and metastatic signaling [44,45]. Proteomic studies have identified statin-induced changes in over 200 proteins across multiple cellular compartments, including mitochondrial respiratory complexes, cytoskeletal proteins, and immune signaling molecules [46,47]. These system-level analyses support the development of biomarker-based personalization strategies, where multi-omics signatures could predict individual patient responses to statin repurposing therapies [47,48]. 

At the mechanistic level, additional studies have shown synthetic lethality to be a key principle underlying statin anticancer effects, where the co-occurrence of mevalonate pathway inhibition and specific genetic alterations leads to selective tumor cell death [49]. Additionally, statins demonstrate significant immunomodulatory properties, reducing key inflammatory markers, including IL-6, TNF-α, and C-reactive protein [50].

### 4.3. Statin Repurposing in Pregnancy: Emerging Therapeutic Opportunities

Recent clinical investigations have explored statin repurposing for obstetric complications, particularly preeclampsia prevention. Pravastatin, with its hydrophilic properties and minimal placental transfer, has undergone Phase II clinical trials for preeclampsia prevention in high-risk pregnancies [51]. The StAmP trial (Statins to Ameliorate early onset Pre-eclampsia) demonstrated promising results, with a 20 mg daily dose of pravastatin showing potential benefits in extending pregnancy duration and reducing maternal–fetal complications [52]. 

The rationale for statin use in pregnancy complications stems from their anti-inflammatory and endothelial protective effects, which may address the underlying pathophysiology of preeclampsia [53]. However, repurposing efforts in pregnancy require particularly rigorous safety considerations, specialized dosing protocols, and enhanced regulatory oversight due to the vulnerable populations involved [51]. Future research should prioritize long-term safety data for both maternal and fetal outcomes, while exploring optimal dosing strategies for pregnancy-specific indications.

### 4.4. Quality Assessment and Evidence Limitations

The current evidence base exhibits significant heterogeneity in study design, patient populations, and outcome measures. Most preclinical studies lack standardized protocols for statin dosing, treatment duration, and endpoint assessment, limiting the translational relevance of findings [54]. The predominance of retrospective observational studies introduces a substantial risk of confounding by indication, where patients receiving statins may have different baseline characteristics affecting their outcomes [55]. Several methodological concerns merit attention. First, the majority of cancer studies utilized statins at standard cardiovascular doses, rather than optimized oncologic dosing regimens [56]. Second, many studies failed to account for statin heterogeneity, as lipophilic statins (atorvastatin, simvastatin) may exhibit different tissue penetration and cellular effects compared to hydrophilic formulations [57]. Third, the temporal relationship between statin initiation and cancer diagnosis remains poorly characterized across studies, potentially affecting the magnitude of observed benefits [56,58].

### 4.5. Evidence Level Assessment and Clinical Applicability

The systematic evaluation of statin repurposing evidence reveals significant limitations when assessed according to established evidence-based medicine hierarchies. Current evidence consists predominantly of the results of Level II–IV studies, with the majority representing hypothesis-generating research rather than definitive clinical guidance.

#### 4.5.1. Evidence Level Classification

Level I Evidence (Randomized Controlled Trials): No completed randomized controlled trials have definitively established the efficacy of statins for any non-cardiovascular indication. The ongoing Budillon et al. VESPA trial represents the highest-quality prospective investigation, but results are not yet available [17].

Level II Evidence (Cohort Studies): Three retrospective cohort studies provide the strongest current clinical evidence: Chen et al. for prostate cancer *(n* = 22,110), Hagiwara et al. for renal cell carcinoma (*n* = 45), and Lohinai et al. for small-cell lung cancer (*n* = 876). While these studies demonstrate associations between statin use and improved outcomes, they are subject to confounding by indication and selection bias [26,27,28].

Level III Evidence (Case Series): The Hungarian regulatory experience provides five individual case reports, which represent the lowest level of clinical evidence, suitable only for hypothesis generation in rare conditions.

Level IV Evidence (Preclinical Studies): The majority of included studies (58%) represent preclinical investigations using in vitro cell culture or animal models [18,19,20,21,22,23]. While these studies provide valuable mechanistic insights, their translational relevance remains uncertain.

#### 4.5.2. Clinical Applicability Assessment

Off-label prescribing based on current evidence should be limited to exceptional cases where no standard therapeutic alternatives exist, and should follow appropriate regulatory frameworks. The evidence base, while promising for future research directions, does not support routine off-label statin use in oncology or infectious diseases.

Prospective observational studies comparing outcomes in patients receiving statins for cardiovascular indications versus matched controls represent a practical research strategy that could provide valuable real-world evidence while avoiding the ethical and economic challenges of randomized trials. The Lohinai et al. study exemplifies this approach, where patients receiving statins for cardiovascular reasons showed improved survival outcomes in small-cell lung cancer [28]. This methodology could be systematically applied across multiple cancer types and healthcare systems to build a more robust evidence base.

### 4.6. Regulatory and Clinical Translation Challenges

The pathway from preclinical promise to clinical implementation faces substantial regulatory and commercial barriers [59,60]. Current regulatory frameworks require robust evidence of efficacy and safety for new indications, even when repurposing well-characterized drugs [5].The absence of patent protection for most statins reduces commercial incentives for expensive clinical trials, creating a “valley of death” between promising preclinical findings and clinical validation [61]. International regulatory approaches to off-label prescribing vary considerably, as exemplified by the Hungarian experience detailed in this review. While some jurisdictions permit off-label use based on physician judgment and available evidence, others require formal regulatory approval through named-patient programs or temporary use authorizations [8]. These regulatory inconsistencies impede the systematic evaluation and implementation of statin repurposing strategies.

### 4.7. Emerging Clinical Evidence and Ongoing Trials

Several high-quality clinical trials are currently addressing the gap between preclinical promise and clinical evidence. The MASTER trial represents a landmark Phase III investigation randomizing 3360 patients with early-stage estrogen receptor-positive breast cancer to receive atorvastatin 80 mg daily versus a placebo for two years [62]. This trial’s primary endpoint of invasive disease-free survival will provide definitive evidence regarding statin efficacy in breast cancer prevention. Recent meta-analyses have strengthened the evidence base for the benefits of statins in breast cancer, demonstrating 19% reductions in both cancer-specific mortality and recurrence risk. Notably, lipophilic statins showed superior efficacy compared to hydrophilic formulations, supporting mechanism-based selection strategies [57].

### 4.8. Safety Considerations and Risk–Benefit Assessment

The well-established safety profile of statins in cardiovascular medicine provides a favorable foundation for repurposing initiatives. However, off-label applications may involve different patient populations, dosing regimens, or treatment durations that could alter the risk–benefit calculus [63]. Recent systematic evaluations of statin therapy in COVID-19 patients revealed no significant clinical benefits from randomized controlled trials, despite observational studies suggesting protective effects [64]. The potential for drug interactions and adverse effects requires careful consideration in cancer patients receiving complex multi-drug regimens [63]. Additionally, the immunomodulatory effects of statins, while potentially beneficial in cancer, might pose risks in immunocompromised patients or those receiving immunosuppressive therapies [50].

## 5. Conclusions

This critical appraisal reveals a complex landscape of preclinical promise tempered by limited high-quality clinical evidence for statin repurposing in non-cardiovascular indications. The current evidence base consists predominantly of early-stage investigations that are hypothesis-generating rather than clinically definitive.

Key findings include the following:

- No completed randomized controlled trials support routine off-label statin use.
- Observational studies suggest potential benefits in specific cancer types, but are subject to significant confounding.
- Mechanistic studies provide valuable insights, but require clinical validation.
- Regulatory frameworks vary widely, necessitating careful adherence to local guidelines.

Research priorities that emerge from this analysis include the following: (1) prospective observational studies comparing outcomes in patients receiving statins for cardiovascular indications versus matched controls; (2) biomarker-guided clinical trials to identify patients that are most likely to benefit from statin repurposing; (3) mechanistic studies to optimize dosing and identify predictive factors; and (4) health economic evaluations to assess cost-effectiveness compared to standard care.

Clinical implications: Off-label statin prescribing should be reserved for exceptional cases where no standard therapeutic alternatives exist, should follow appropriate regulatory frameworks, and should be accompanied by careful monitoring and reporting. Recommendations for routine off-label use cannot be supported by current evidence.

Future research should focus on generating high-quality clinical evidence through pragmatic study designs, while maintaining appropriate ethical and regulatory standards. The low cost and established safety profile of statins make them attractive candidates for repurposing, but this potential must be realized through rigorous scientific investigation, rather than premature clinical adoption.

## Figures and Tables

**Table 1:** The regulatory frameworks for off-label prescriptions across regions.

Region	Regulatory Authority	Off-Label Approval Mechanism
**USA**	U.S. Food and Drug Administration (FDA)	Physician’s discretion; no promotion allowed; informed consent advised [5]
**Europe**	European Medicines Agency (EMA)	Member states regulated under Directive 2001/83/EC; no EU-wide process [6]
Germany	Federal Institute for Drugs and Medical Devices (BfArM)	Case-by-case basis with patient consent; insurer review possible [10]
Italy	Italian Medicines Agency (AIFA)	AIFA-approved list under Law 648/1996; also compassionate use [7]
Hungary	National Institute for Public Health and Pharmacy (NNGYK)	Named-patient request system via NNGYK when no therapeutic alternatives exist [13]
France	Agence Nationale de Sécurité du Médicament (ANSM)	RTU framework; aligned with Public Health Code and 2021 reforms [9]
**China**	National Medical Products Administration (NMPA)	Permitted under 2022 Physicians Law; justification and patient consent required [11]

**Table 2:** Evidence-based classification of statin repurposing studies identified through systematic literature analysis. Section A: Theoretical evidence (study protocols). Section B: Preclinical evidence—B1: In vitro studies; B2: Animal model studies; B3: Mixed preclinical/clinical studies. Section C: Clinical evidence (observational studies).

Section A: Theoretical Evidence (Study Protocols)							
Author (Year)	Indication	Category	Study Design	Statin Type	Outcome/Effect	Notes/Role	Patient Number
Budillon et al. NCT05711095 (2024) [17]	Metastatic pancreatic cancer	Oncology	Phase II RCT protocol	Simvastatin	Hypothesized improvement in PFS (protocol study—no results reported)	Protocol study demonstrating research interest in statin repurposing	240 planned (study ongoing)
**Section B1: Preclinical evidence: In Vitro Studies**							
Gaber et al. (2024) [18]	Breast cancer	Oncology	In vitro + in vivo (mice)	Atorvastatin	↑ apoptosis, tumor inhibition	Drug delivery-enhanced model	No human patients (in vitro only)
Irie et al. (2024) [19]	Melanoma	Oncology	In vitro (human/canine)	Atorvastatin	↑ cytotoxicity with dipyridamole	Supports drug combination approach	No human patients (cell lines only)
Kobayashi et al. (2022) [20]	Ovarian cancer	Oncology	In vitro + patient-derived samples	Simvastatin	Biomarkers identified for statin responders	Precision oncology repositioning	Clinical sample analysis, N = unknown
**Section B2: Preclinical evidence: Animal Model Studies**							
Author (Year)	Indication	Category	Study Design	Statin Type	Outcome/Effect	Notes/Role	Patient Number
Juneja et al. (2017) [21]	Colorectal cancer	Oncology	In vitro + mouse xenograft	Lovastatin	↓ metastasis via MACC1 inhibition	Metastasis suppression model	No human patients (murine models)
Ribeiro et al. (2017) [22]	Cryptococcosis	Infectious disease	In vitro + in vivo (mice)	Atorvastatin	↑ survival + ↓ fungal burden with fluconazole	First in vivo antifungal repositioning data	Mice model, N = 6 per group
Palacios-Rápalo et al. (2023) [23]	Dengue virus infection	Infectious disease	In vitro + in vivo (mice)	Atorvastatin	↓ DENV replication with ivermectin combo	Novel antiviral mechanism	Mouse model studies
**Section B3: Preclinical evidence: Mixed Preclinical/Clinical Studies**							
Author (Year)	Indication	Category	Study Design	Statin Type	Outcome/Effect	Notes/Role	Patient Number
Fuentes-Fayos et al. (2023) [24]	Glioblastoma	Oncology	In vitro + in vivo + human cohort	Simvastatin	Senescence, ↓ proliferation	Translational study with multi-level evidence	Small clinical subgroup (preclinical focus)
Duarte et al. (2021) [25]	COVID-19	Infectious disease	Organoids + transcriptomics	Atorvastatin	↓ viral entry; ↑ immune modulation	Target for pandemic preparedness	No human patients (organoid model)
**Section C: Clinical Evidence (Observational Studies)**							
Author (Year)	Indication	Category	Study Design	Statin Type	Outcome/Effect	Notes/Role	Patient Number
Chen et al. (2018) [26]	Prostate cancer	Oncology	Retrospective cohort	Simvastatin, Lovastatin	↓ mortality (HR = 0.63 with high DDD)	Large-scale population data	22,110
Hagiwara et al. (2018) [27]	Renal cell carcinoma	Oncology	Retrospective + mechanistic validation	Simvastatin	↑ efficacy of mTOR inhibitors	Clinically relevant with mechanistic link	Retrospective cohort, N = 45
Lohinai et al. (2016) [28]	Small-cell lung cancer	Oncology	Retrospective (Hungarian)	Multiple statins	↑ survival with statin use	Direct Hungarian evidence	876

↑: increased, ↓: decreased.

**Table 3:** Summary of Hungarian off-label use requests for statins [29].

Year	Patient Demographics	Indication	Statin Type and Dose	Proposed Dosage and Expected Duration of Therapy	Approval Conditions
2012	Female child, 4 years old	Hypercholesterinaemia	Simvastatin, 10 mg	5 mg daily, increased to a maximum of 20 mg/day, continuous use	Expert opinion; post-treatment evaluation report
2017	Male infant, 1.5 months old	Smith–Lemli–Opitz syndrome	Simvastatin, 10 mg	0.5 mg/kg/day, continuous use	Literature support; 3-month reporting
2021	Male, 64 years old	Actinic porokeratosis	Simvastatin, 40 mg	Topical ointment applied twice daily, for 3 months	Clinical/safety monitoring; report submission
2024	Male, 49 years	Superficial disseminated porokeratosis	Simvastatin, 40 mg	Simvastatin: 2% topical cream, once daily, continuously or in cycles depending on status	Post-treatment evaluation report
2025	Female, 55 years	Actinic porokeratosis	Simvastatin, 40 mg	Apply thinly twice daily on affected area—topical application (external use)	Post-treatment evaluation report

**Table 4:** Summary of key oncology-focused statin repurposing studies.

Author (Year)	Indication	Study Design	Statin	Treatment Approach	Key Effect	Relevance	Strength	Weakness	Applicability
Budillon et al. (2024) [17]	Metastatic pancreatic adenocarcinoma	Prospective clinical trial protocol (Phase II RCT)	Simvastatin (20 mg/day)	Combination with VPA + chemo	Hypothesized ↑ PFS; planned translational biomarker endpoints	Clinical/translational	Randomized design; translational endpoints; known safety profiles	Protocol only—no outcome data yet; limited to PDAC	Potential adjunct to first-line chemotherapy
Chen et al. (2018) [26]	Prostate cancer	Retrospective cohort study (national database)	Simvastatin, Lovastatin	Monotherapy	↓ Prostate cancer mortality (HR 0.63 for >180 DDD/year)	Clinical epidemiologic	Large sample; clear dose–response; robust adjustment for confounders	Observational; residual confounding; Taiwan-only population	Potential chemopreventive use in hormone-driven cancers
Fuentes-Fayos et al. (2023) [24]	Glioblastoma multiforme	Retrospective cohort + in vitro + in vivo xenografts	Simvastatin	Combination with Metformin, adjunctive	Synergistic with metformin: ↑ apoptosis, senescence, ↑ OS in cohort	Translational	Multi-tiered (human, cell, animal); mechanistic depth	Retrospective design; small clinical subgroup; intratumoral dosing	Adjunctive strategy with metformin in GBM therapy
Gaber et al. (2024) [18]	Breast cancer	In vitro (MCF-7 cells) + in vivo (Ehrlich mice)	Atorvastatin (SLN, Lf-SLN)	Formulation	↑ tumor cell apoptosis; ↓ tumor volume (SLN > free drug)	Translational/mechanistic	Novel targeted delivery; multiple efficacy measures	Preclinical only; IP delivery; single-tumor model; no PK data	Basis for nanoparticle-enhanced statin therapy in BC
Hagiwaraa et al. (2018) [27]	Renal cell carcinoma	Retrospective (everolimus users) + in vitro + xenograft	Simvastatin	Combination with Everolimus	↑ PFS (7.5 vs. 3.2 months); ↑ mTOR-inhibitor efficacy	Translational	Real-world + mechanistic concordance; Rb activation elucidated	Retrospective; potential bias among statin users; single center	Rationale for statin + mTOR inhibitor combination in RCC
Juneja et al. (2017) [21]	Colorectal cancer	In vitro + in vivo mouse xenograft (MACC1+)	Lovastatin	Monotherapy, preclinical	↓ Metastasis via MACC1 downregulation	Mechanistic/translational	Clear target (MACC1); robust functional assays	Preclinical; limited to MACC1-driven tumors; IP dosing	Prototype for targeting metastasis-associated pathways
Irie et al. (2024) [19]	Melanoma	In vitro (human and canine cell lines)	Atorvastatin	Combination with Dipyridamole	Synergistic growth inhibition with dipyridamole	Mechanistic	Demonstrates feedback blockade; cross-species consistency	No in vivo validation; high in vitro doses; safety unknown	Suggested combination therapy strategy for melanoma
Kobayashi et al. (2022) [20]	Ovarian cancer	In vitro + ex vivo drug response in patient tissue	Simvastatin	Biomarker-guided	Biomarker-guided sensitivity; synergy with paclitaxel/panobinostat	Translational/mechanistic	Integrated biomarker discovery + synergy testing	Preclinical/ex vivo only; requires clinical validation	Precision medicine statin regimens in ovarian cancer
Lohinai et al. (2016) [28]	Metastatic small-cell lung cancer (SCLC)	Retrospective clinical cohort (Hungarian SCLC data)	Various (atorva/simva)	Monotherapy, observational	↑ OS (8.4 vs. 6.1 months) in statin users	Clinical	Large real-world cohort; multivariate adjustment	Retrospective; selection bias; lack of dose/duration details	Low-cost adjunct in SCLC—warrants prospective study

↑: increased, ↓: decreased.

**Table 5:** Summary of key infectious disease-focused statin repurposing studies.

Author (Year)	Indication	Study Design	Statin	Treatment Approach	Key Effect	Relevance	Strength	Weakness	Applicability
Ribeiro et al. (2017) [22]	Cryptococcal infection	In vitro + in vivo (murine model)	Atorvastatin	Monotherapy + fluconazole	↓ fungal burden in lungs; synergy with fluconazole	Translational	Dual model; synergistic antifungal effect; dose-effective	Lack of clinical data; unclear PK translatability; no detailed mechanistic confirmation	Promising adjunct therapy for fungal infections; needs clinical validation
Duarte et al. (2021) [25]	SARS-CoV-2 infection	In silico + in vitro (lung organoids)	Atorvastatin	Organoid model	↓ viral entry via lipid raft modulation; no cytotoxicity	Translational	Human lung organoids; multimodal design (computational + lab)	No in vivo or clinical validation; pseudotyped virus used; no pharmacokinetic assessment	Basis for antiviral trials in early COVID-19 intervention
Palacios-Rápalo et al. (2023) [23]	Dengue virus (DENV) infection	In vitro (Huh-7 cells) + in vivo (AG129 mice)	Atorvastatin	Monotherapy + ivermectin	↓ viral replication, cytokines, mortality; ↑ effect with ivermectin combination	Translational	Demonstrated statins alone and in combo; mechanistic insight into nuclear transport disruption	Immunodeficient model; no PK or human data; NS3 nuclear transport not evaluated in vivo	Foundation for combination therapy trials in viral infections

↑: increased, ↓: decreased.

## Data Availability

The original contributions presented in this study are included in the article. Further inquiries can be directed to the corresponding author.

## Abbreviations

The following abbreviations are used in this manuscript:

AIDS	Acquired Immunodeficiency Syndrome
BBB	Blood–Brain Barrier
CA 19-9	Carbohydrate Antigen 19-9
CCL	Chemokine Ligand
CI	Confidence Interval
COVID-19	Coronavirus Disease 2019
CRC	Colorectal Cancer
DCR	Disease Control Rate
DDD	Defined Daily Dose
DENV	Dengue Virus
EMA	European Medicines Agency
FDA	Food and Drug Administration
GBM	Glioblastoma Multiforme
HDAC	Histone Deacetylase
HR	Hazard Ratio
HMGCR	3-Hydroxy-3-Methylglutaryl-Coenzyme A Reductase
IC_50_	Half Maximal Inhibitory Concentration
IFN-γ	Interferon Gamma
IL	Interleukin
mCRC	Metastatic Colorectal Cancer
mPDAC	Metastatic Pancreatic Ductal Adenocarcinoma
mTOR	Mammalian Target of Rapamycin
NFκB NNGYK	Nuclear Factor Kappa B National Institute of Public Health and Pharmacy
NS5	Nonstructural Protein 5
ORR	Objective Response Rate
OS	Overall Survival
PAXG	Nab-paclitaxel, Gemcitabine, Capecitabine, Cisplatin
PCa	Prostate Cancer
PFS	Progression-Free Survival
PRISMA	Preferred Reporting Items for Systematic Reviews and Meta-Analyses
RCC	Renal Cell Carcinoma
SASP	Senescence-Associated Secretory Phenotype
SCLC	Small-Cell Lung Cancer
SREBP2	Sterol Regulatory Element-Binding Protein 2
TNF-α	Tumor Necrosis Factor Alpha
VPA	Valproic Acid
YAP	Yes-Associated Protein
